# Case Report: Efgartigimod as an additional therapy for MOG antibody-associated disease overlapping GFAP-IgG

**DOI:** 10.3389/fimmu.2025.1624660

**Published:** 2025-11-17

**Authors:** Yun Zhu, Juanjuan Zhang, Hongru Li, Ling Wei, Yanghua Tian, Kai Wang

**Affiliations:** 1 Department of Neurology, The First Affiliated Hospital of Anhui Medical University, Hefei, Anhui, China; 2 Department of Neurology, The Second Affiliated Hospital of Anhui Medical University, Hefei, Anhui, China

**Keywords:** myelin oligodendrocyte glycoprotein antibody-associated disease, autoimmune glial fibrillary acidic protein astrocytopathy, efgartigimod, overlapping syndrome, demyelinating disease

## Abstract

**Introduction:**

Myelin oligodendrocyte glycoprotein (MOG) antibody-associated disease (MOGAD) and autoimmune glial fibrillary acidic protein (GFAP) astrocytopathy have received increasing attention in recent years. However, the coexistence of anti-MOG and anti-GFAP antibodies has rarely been reported.

**Case:**

A 53-year-old man presented with a headache, slow reaction, nonsense talk, unsteady walking without diplopia or decreased vision. Lumbar puncture revealed the presence of anti-MOG and anti-GFAP antibodies in the cerebrospinal fluid. Magnetic resonance imaging revealed multiple high signal intensities in the white matter. The patient was diagnosed with MOGAD syndrome with overlapping GFAP-IgG. Treatment comprised high-dose methylprednisolone and efgartigimod therapy, followed by gradual tapering of oral prednisolone and the addition of an immunosuppressant, leading to symptomatic improvement and sustained remission.

**Conclusion:**

We report a case of MOGAD-overlapping GFAP IgG treated with combination therapy of steroids and efgartigimod. This case enhances our understanding of the clinical manifestations of overlapping syndromes and expands the treatment options for this disorder.

## 1 Introduction

Myelin oligodendrocyte glycoprotein (MOG) antibody-associated disease (MOGAD) and autoimmune glial fibrillary acidic protein (GFAP) astrocytopathy are demyelinating diseases of the central nervous system (CNS) that have received increasing attention in recent years. MOGAD is defined as an autoantibody targeting MOG, a surface glycoprotein in CNS myelin (1). Predominantly the IgG1 subtype, these antibodies drive pathogenesis through complement activation, antibody-dependent cellular cytotoxicity (ADCC), and myelin sheath disruption. This inflammatory cascade recruits CD4 + T cells, macrophages, and neutrophils, resulting in demyelination, with relative axonal preservation (2). In contrast, GFAP astrocytopathy (GFAP-A) is an autoimmune disorder targeting astrocytes, defined by GFAP—an intracellular cytoskeletal component. Although GFAP-IgG is a specific CSF biomarker, its direct pathogenicity remains unclear, and it may in fact be accompanied by an as yet unidentified pathogenic autoantibody targeting the astrocytic plasma membrane (3). Histopathological analyses revealed extensive perivascular CD8 + T cell infiltration with astrocytic loss, indicating that cytotoxic T lymphocytes drive the core pathogenesis (4). Both conditions commonly manifest as optic neuritis, encephalomeningitis, brainstem encephalitis and myelitis. However, the coexistence of anti-MOG and anti-GFAP antibodies has rarely been reported. Currently, fewer than 200 cases of MOGAD overlapping with GFAP-IgG have been reported worldwide, which is significantly fewer than those of isolated MOGAD or GFAP astrocytopathy. Patients with dual antibody positivity often present with a wide spectrum of neurological symptoms, encompassing the core manifestations of both conditions, including fever, headache, altered consciousness, psychiatric/behavioural abnormalities, seizures, visual impairment, bladder/bowel dysfunction, sensory deficits, and limb weakness (5). Owing to the striking similarities in clinical manifestations, neuroimaging features, and laboratory findings between MOGAD and GFAP-A, early diagnosis poses significant challenges. The current acute-phase treatments primarily consist of high-dose steroid pulse therapy, intravenous immunoglobulin (IVIG), and plasma exchange (PE). Most patients respond to steroids (5). Efgartigimod is an FcRn-targeting immunomodulator that blocks FcRn-IgG binding, accelerates pathogenic autoantibody degradation, and reduces systemic IgG levels. FDA approved in 2021 for anti-AChR+ myasthenia gravis, it is a novel therapeutic option for antibody-mediated disorders, including immune thrombocytopenia, autoimmune encephalitis, CIDP, and NMOSD (6–9). Herein, we present a single adult case of MOGAD overlapping with GFAP-IgG that was treated with efgartigimod.

## 2 Case description

A 53-year-old male presented with a 6-day history of cognitive slowing, disorganised speech, gait instability, and headaches without fever or additional neurological or systemic symptoms. Neurological examination was conducted, and the results for cranial nerve examination and Babinski sign were negative. The patient was unable to cooperate during the examination of higher cognitive function. He was admitted to our hospital on 3 April. Before admission, the patient had been treated at a local hospital, and laboratory assessments conducted on 31 March indicated elevated white blood cell (WBC) counts and inflammatory markers. The patient’s pre-admission white blood cell count was 10.54×10^9^/L, with neutrophils at 8.09×10^9^/L. Cranial CT indicated bilateral symmetrical, low-density areas surrounding the lateral ventricles, suggesting ischaemic changes. Diffusion-weighted imaging (DWI) did not reveal any abnormalities. During this period, his symptoms progressively deteriorated and were characterised by confusion towards family members and inappropriate responses to queries, which were notably exacerbated at night.

## 3 Diagnostic assessment

**Figure 1:**
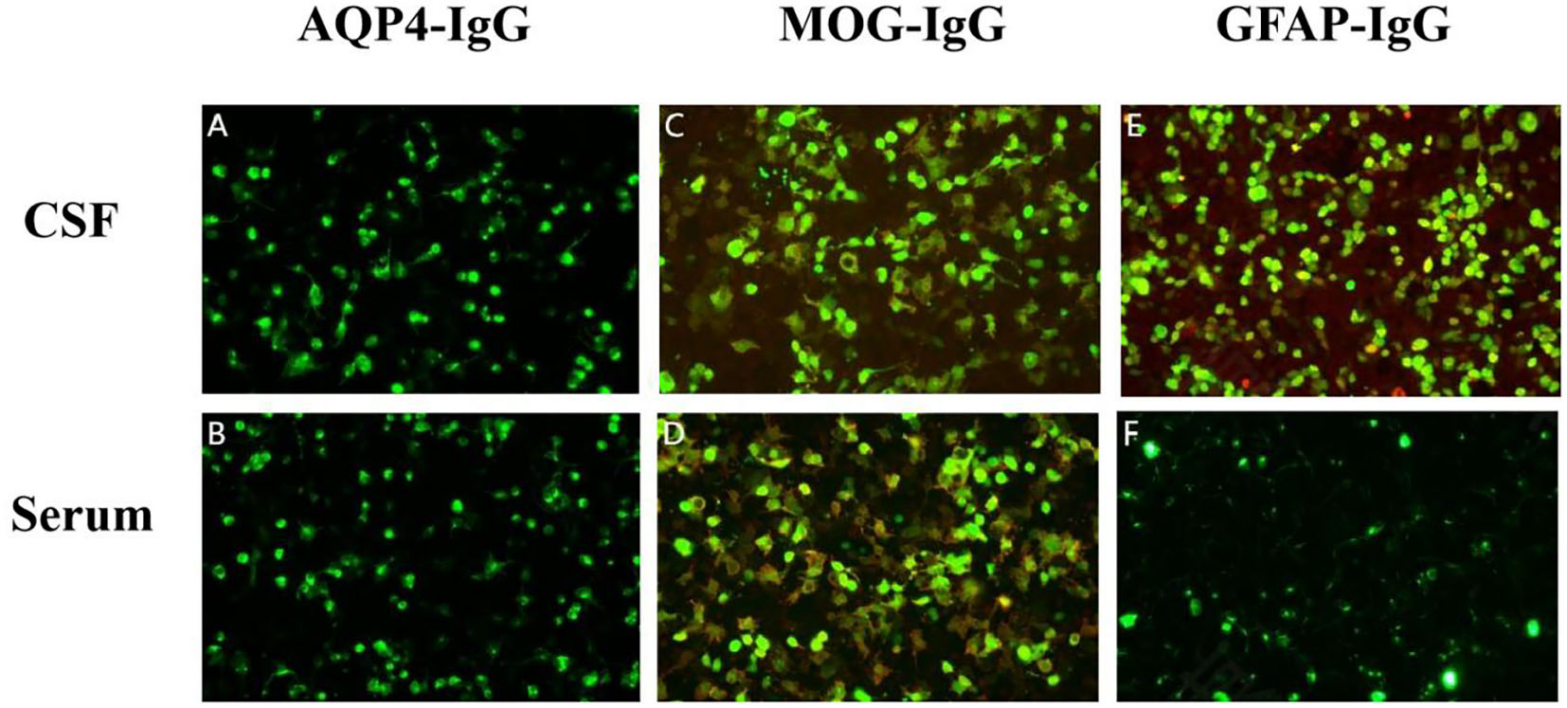
Detection of autoimmune antibodies using cell-based assays: negative AQP4-IgG in cerebrospinal fluid (CSF) **(A)** and serum **(B)**; positive myelin oligodendrocyte glycoprotein (MOG)-IgG in CSF [**(C)**, titer 1:10] and serum [**(D)**, titer 1:10]; and positive glial fibrillary acidic protein (GFAP)-IgG only in CSF [**(E)**, titer 1:1] but negative in serum **(F)**. The antibodies in serum were further confirmed by Tissue-Based Assay (TBA) with rat brain and kidney tissue (1:100).

After admission, a lumbar puncture was performed on 3 April which revealed an intracranial pressure of 250 mm H_2_O. Cerebrospinal fluid (CSF) analysis revealed a normal glucose level of 2.22 mmol/L with increased protein (2.51 g/L). There were 422×10^6^/L leukocytes, with 34% monocytes and 66% polykaryocytes. The serum IgG level was 7.33 g/L. Etiological studies were negative, including acid-fast staining, fungal and bacterial smears, and ink staining (CSF cytology showed a mixed cell reaction). NMDAR-IgG, LGI1-IgG, CASPR2-IgG, GABABR-IgG, AMPAR1-IgG, AMPAR2-IgG, IgLON5-IgG, DPPX-IgG, GAD65-IgG, mGluR5-IgG, GlyR-IgG, and D2R-IgG were negative in both serum and CSF. WBC count was 21.99×10^9^/L. The serum glucose level in the serum was 5.61 mmol/L. The erythrocyte sedimentation rate (ESR) increased to 56 mm/h. Tests for antinuclear antibodies (ANA), ANCA, anticardiolipin antibodies (ACL), syphilis, and HIV were all negative. Tumour markers such as AFP, CEA, CA 19-9, CA 125, CA 72-4, CYFRA 21-1, FER, and CA 50 were negative. Chest CT showed fibrosis and small nodules. Based on the elevated CSF leukocytes and psychiatric symptoms, the patient was diagnosed with suspected viral encephalitis and was initiated on intravenous ganciclovir 350 mg every 12 h for antiviral therapy. Further testing for demyelinating antibodies showed MOG-IgG positivity in both the serum and CSF, with titres of 1:10 (**Figures 1C, D**). Additionally, GFAP-IgG was detected in the CSF at a titre of 1:1 (**Figure 1E**), whereas serum testing was negative for GFAP-IgG (**Figure 1F**). Both the serum and CSF tested negative for AQP4-IgG (**Figures 1A, B**). Therefore, the patient was diagnosed with MOGAD-overlapping GFAP IgG antibodies. After testing positive for MOG and GFAP antibodies, the patient underwent high-dose steroid pulse therapy. The regimen consisted of methylprednisolone 500 mg/day via IV infusion for 5 days, followed by 250 mg/day IV for 3 days, 120 mg/day IV for 3 days, and 60 mg/day orally for 3 days. Owing to the lack of significant improvement in symptoms one day after the use of methylprednisolone, 800 mg of efgartigimod was administered on 5 April with full informed consent from the patient’s family. On the same day, he developed glossoptosis with deterioration of consciousness due to stupor. Arterial blood gas analysis revealed unstable oxygen saturation. He was transferred to the ICU for further monitoring until 10 April. During ICU treatment, the level of consciousness gradually recovered, and oxygen saturation gradually stabilised under nasal catheter oxygen inhalation.

**Figure 2:**
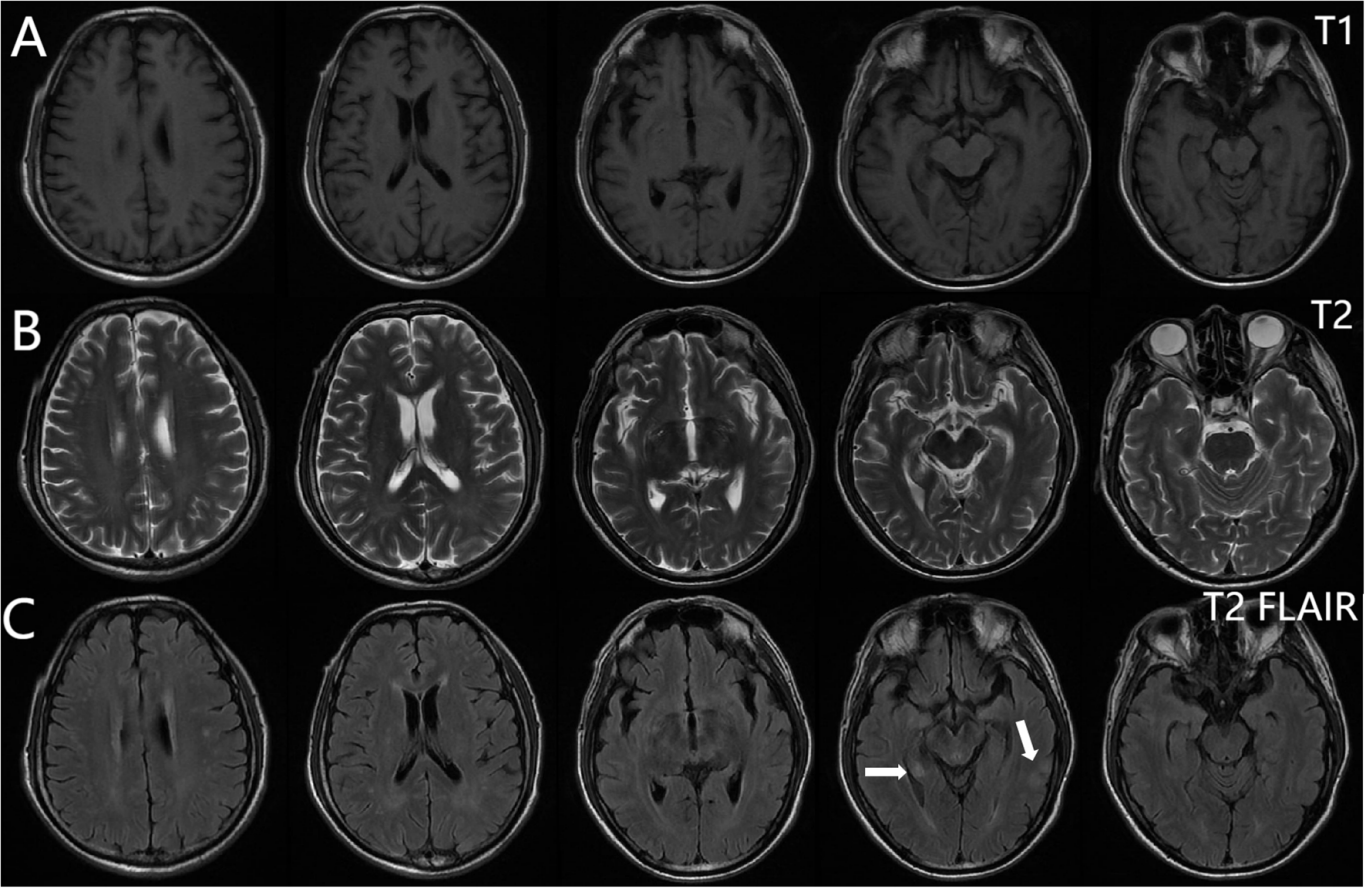
Brain magnetic resonance imaging. T1-weighted **(A)**, T2-weighted **(B)**, T2 FLAIR **(C)**. Multiple punctate lesions with T1 hypointensity, T2 hyperintensity, and FLAIR hyperintensity in the centrum semiovale, periventricular white matter, and basal ganglia regions.

**Figure 3:**
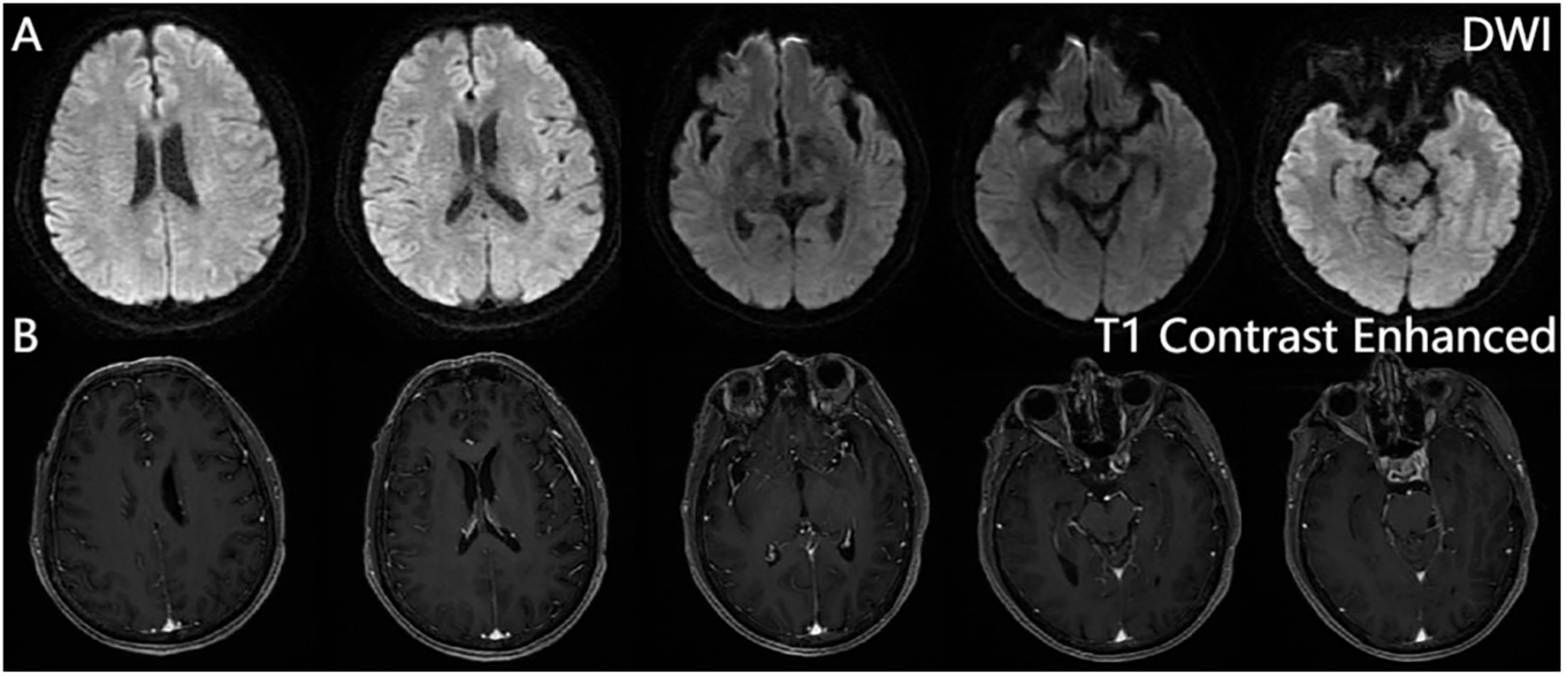
Brain magnetic resonance imaging. Diffusion-weighted imaging (DWI) **(A)**, contrast-enhanced sequences **(B)**. No significant hyperintensity is observed on DWI, and post-contrast sequences reveal no abnormal enhancement in the brain parenchyma, calvarium, or meninges.

**Figure 4:**
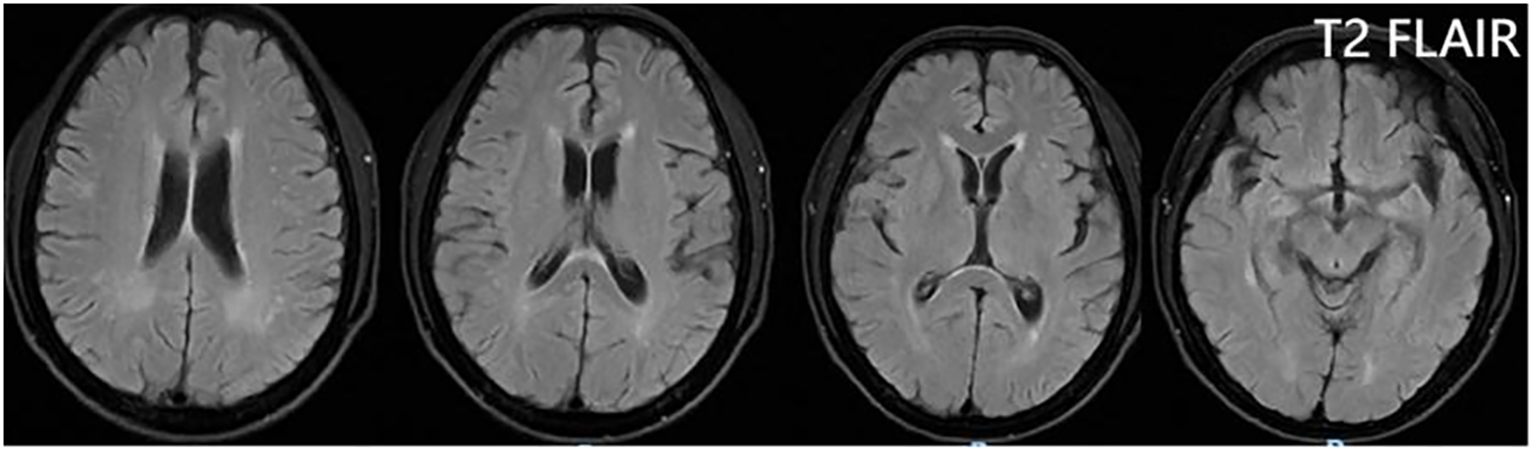
T2 FLAIR showing that the multiple hyperintensities in the medial temporal lobe and temporal cortex became small three months after discharge.

From 8 April, the clinical symptoms demonstrated progressive improvement, marked by the patient’s transition from stupor to drowsiness on a consciousness scale. By 16 April the patient was alert and attentive to these questions. A high dose of methylprednisolone was administered for 5 days and the dose was subsequently decreased gradually. Dehydration was implemented to reduce intracranial pressure. Another lumbar puncture was performed on the tenth day of admission (15 April). CSF analysis revealed normal glucose levels with elevated protein (0.89 g/L). The leukocyte count in the CSF was 78×10^6^/L, representing a significant decrease compared to the WBC count in the CSF on 4 April. MOG-IgG in the CSF and serum was negative, and GFAP-IgG in the CSF was negative. The serum IgG decreased to 3.21 g/L, reflecting a 56% reduction compared with the previous serum IgG level. Another cranial MRI was performed on 3 April. The T2 FLAIR showed multiple hyperintensities in the posterior horn of the lateral ventricle, medial temporal lobe, and temporal cortex (**Figures 2**, **3**). After tapering the corticosteroid therapy, the patient was transitioned to oral administration. He was discharged from the hospital on 18 April with a notable improvement in symptoms. At the one-month post-discharge outpatient follow-up visit, all psychiatric symptoms had disappeared completely. Treatment included low-dose oral steroids and mycophenolate mofetil. Three months after discharge, the patient underwent cranial MRI, which showed that the multiple hyperintensities in the medial temporal lobe and temporal cortex had decreased (**Figure 4**). Ten months after the patient’s initial presentation, no symptoms except for cognitive impairment were observed, and no drug-related adverse reactions were reported, with the patient reporting an “irritable mood” (MMSE: 26, MOCA: 13, HAMA: 5, HAMD: 1). The impaired dimensions included visual-spatial function, execution, and memory.

## 4 Discussion

The core pathogenesis of MOGAD centers on pathogenic anti-MOG-IgG1 antibodies binding to the MOG antigen on the surface of oligodendrocytes. Through pathways such as CDC, ADCC, and phagocytosis (ADCP), this binding leads to oligodendrocyte damage, inflammatory responses, demyelination, and secondary axonal injury. In MOGAD patients, cytokines associated with Th17, Treg, and Th1 cells are upregulated in the CSF. Furthermore, in animal models, MOG-specific CD4+ T cells participate in inflammatory disorders of the central nervous system (10, 11). The core pathogenesis of GFAP astrocytopathy involves pathogenic anti-GFAP IgG antibodies (predominantly IgG1 subtype) targeting and binding to GFAP in astrocyte foot processes. This binding directly damages astrocytes through CDC and ADCC. Concurrently, it triggers CD8+ T-cell infiltration and the release of pro-inflammatory cytokines (such as IFN-γ and IL-17), initiating an inflammatory cascade (12). This cascade results in blood-brain barrier disruption, perivascular “radial” inflammatory infiltration (characteristically manifesting as perivascular enhancement on MRI), and astrocyte dysfunction. Approximately 50% of cases are associated with tumors (e.g., ovarian teratomas), suggesting paraneoplastic cross-immunity contributes to autoantibody production (13). Ultimately, this process leads to targeted astrocyte injury mediated by the synergistic actions of humoral and cellular immunity. To date, no studies have investigated the co-operative mechanisms of MOG and GFAP antibodies. Therefore, the following hypotheses are based on current evidence. In this case, the MOG and GFAP antibodies jointly compromise the blood-brain barrier, allowing a significant influx of inflammatory cytokines and immune cells into the central nervous system. This may lead to more severe symptoms. Concurrent damage to oligodendrocytes and astrocytes accelerates neural injury. Furthermore, cell death releases additional autoantigens, expanding autoimmune targets and making the disease more challenging to control.

The typical clinical features of MOGAD and autoimmune GFAP astrocytopathy include optic neuritis, encephalomeningitis, brainstem encephalitis and myelitis (14–17). Coexisting GFAP and MOG syndromes are rare. Fang summarised 18 reported cases of overlapping MOGAD and autoimmune GFAP astrocytopathy (18). Among the 14 cases with sufficient clinical data, including seven adults and seven children, the predominant clinical manifestation was meningoencephalomyelitis, followed by meningoencephalitis (18). Most patients received high-dose methylprednisolone therapy at the early stage, yielding a favourable response (18). Treatments include high-dose steroid therapy, IVIG therapy, plasma exchange, and rituximab therapy (14–18).

**Figure 5:**
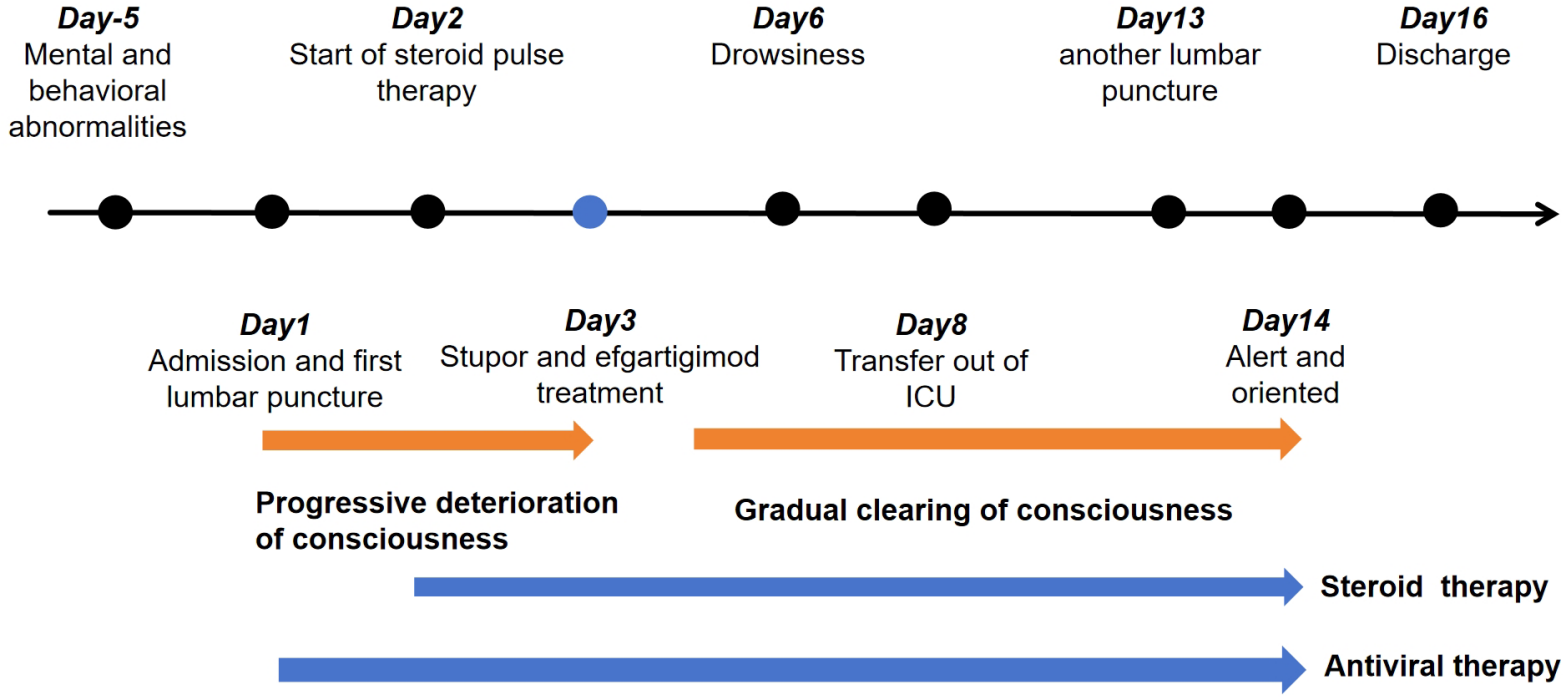
Timeline of the patient’s entire course of treatment.

Our patient exhibited encephalopathy characterised by psychiatric and behavioural abnormalities. T2 flair on cranial MRI revealed multiple white matter hyperintensities. CSF analysis revealed increased leukocyte and protein levels. Despite the initial misdiagnosis of a CNS infection, prompt initiation of immune therapy ensued, following the detection of MOG-IgG and GFAP-IgG. Treatments included traditional high-dose steroids and efgartigimod (**Figure 5**). Efgartigimod lowers IgG levels by specifically binding to the neonatal Fc receptor (FcRn), which expresses on vascular endothelial cells and certain immune cells, primarily protects IgG antibodies from lysosomal degradation, recycling them and maintaining their long half-life (~3 weeks). Efgartigimod is an FcRn antagonist designed with high affinity to bind FcRn competitively. This binding blocks the interaction of endogenous IgG with FcRn. IgG unable to bind FcRn cannot be “recycled” and is instead directed toward lysosomal degradation. The result is a significant, rapid, and reversible reduction in the overall levels of all IgG subtypes (including pathogenic autoantibodies) in the circulation (typically by about 60-75%). Efgartigimod accelerates the catabolism of all IgGs (including pathogenic autoantibodies) by blocking the FcRn-mediated IgG recycling pathway, thereby reducing their serum concentrations. This mechanism theoretically offers therapeutic potential for MOGAD and GFAP-IgG related astrocytopathy, both mediated by IgG1-type antibodies, by aiming to reduce pathogenic antibody load and consequently lessen neuroinflammation and damage (19). Currently, efgartigimod is used to manage adult systemic myasthenia gravis with anti-AChR antibodies (6). Therefore, the use of efgartigimod constituted an off-label application. After combination therapy with steroids and efgartigimod, the patient’s symptoms gradually improved. Additionally, MOG-IgG in the CSF and serum, and GFAP-IgG in the CSF were negative. A > 50% reduction in serum IgG levels was observed. The patient did not experience any adverse reactions to efgartigimod. Therefore, efgartigimod was effective and safe for our patient with overlapping MOGAD and autoimmune GFAP astrocytopathy.

MOGAD and GFAP antibody-associated disorders are easily confused with central nervous system (CNS) infections, autoimmune encephalitis, and metabolic encephalopathy. Due to blood-brain barrier disruption and inflammatory responses, patients with MOGAD and GFAP antibody-associated disorders exhibit elevated CSF cell counts and protein levels. Some GFAP antibody-associated disorder patients also have low CSF glucose, necessitating differentiation from CNS infections, particularly tuberculosis (20). Next-generation sequencing (NGS), T-SPOT.TB, and CSF cytology aid in this differential diagnosis (21). The clinical manifestations of MOGAD and GFAP antibody-associated disorders resemble those of autoimmune encephalitis; thus, autoimmune encephalitis antibody testing can help distinguish them (22). Some patients may present with non-convulsive seizures, making VEEG a useful diagnostic tool (23). Metabolic encephalopathy patients can exhibit psychiatric symptoms and cognitive changes, which also requires differentiation from GFAP and MOGAD patients. However, metabolic encephalopathy patients often have underlying factors such as nutritional deficiencies, internal environment imbalances, and abnormal blood glucose. Imaging typically reveals symmetrical intracranial abnormalities (24). Therefore, differentiation can be achieved through medical history, biochemical tests, and imaging studies.

Our study has several limitations. First, the elevated WBC count in the patient’s blood was difficult to explain, given the absence of fever and the lack of infection in the respiratory or urinary system. Considering the obvious increase in leukocytes in the CSF, with 66% polykaryocytes, the possibility of a CNS infection was considered. Combined with an elevated erythrocyte sedimentation rate, these findings suggest a possible infection. We speculate that this infection may have triggered an immune response, resulting in the patient’s positive MOG and GFAP antibodie (25, 26). We explained that the overlapping syndromes of MOGAD and autoimmune GFAP astrocytopathy mimicked infectious encephalitis, a phenomenon previously documented in the literature (17). To enhance the credibility of our findings, the inclusion of metagenomic next-generation sequencing would be valuable for ruling out infectious encephalitis. Additionally, the failure to perform T-SPOT.TB, and cerebrospinal fluid (CSF) culture represents a limitation of the study, as these tests serve as key elements for differential diagnosis. Second, although this patient exhibited low-titer MOG antibody positivity, the diagnosis can be established based on the International MOGAD Panel’s proposed supportive diagnostic criteria (27). In addition, given the patient’s complete recovery after immunotherapy, we maintain the diagnosis of MOGAD that overlapped with GFAP-IgG.

## 5 Conclusion

While high-dose corticosteroids remain first-line for MOGAD with GFAP-IgG comorbidity, this case demonstrates Efgartigimod’s potential as a precision immunotherapy for antibody-driven neuroinflammatory syndromes (28). By enabling simultaneous CNS-penetrant clearance of pathogenic autoantibodies in treatment-refractory patients, it challenges the paradigm of irremediable severe disease. Future trials must validate sustained efficacy and optimize dosing guided by longitudinal antibody tracking—paving the way for targeted management of neuroimmune overlap disorders.

## Data Availability

The original contributions presented in the study are included in the article/supplementary material. Further inquiries can be directed to the corresponding author.
